# Health Benefits of Universal Influenza Vaccination Strategy

**DOI:** 10.1371/journal.pmed.0050216

**Published:** 2008-10-28

**Authors:** Cécile Viboud, Mark Miller

## Abstract

Cécile Viboud and Mark Miller discuss the implications of a new study that used a mathematical model to simulate influenza transmission in nursing homes.

Most countries in Europe and North America target influenza immunization to persons at highest risk for complications, including seniors 65 years and older, those with certain chronic illnesses, and young children. Despite increases in vaccination rates in these high-risk groups over the last few decades, morbidity and mortality from influenza remain high.

## Disappointing Results of Targeted Influenza Vaccination Strategies

**Figure 1:**
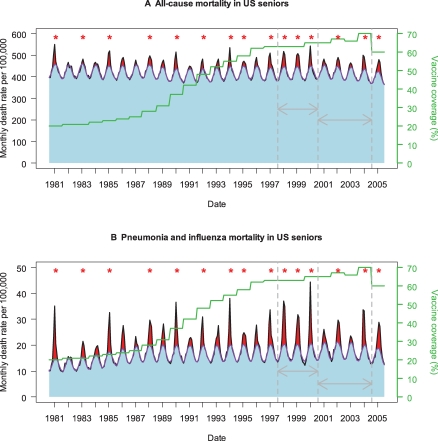
Time Trends in Influenza Vaccine Coverage and Influenza-Related Mortality in People 65 Years and Older in the US, Based on Two Death Categories (A) All-cause mortality. (B) Pneumonia and influenza mortality. The black curve illustrates observed monthly mortality rates, the purple curve represents a monthly model baseline above which mortality is attributed to influenza [1], and the green curve represents trends in seasonal vaccine coverage in people 65 years and older. Red shaded areas represent seasonal estimates of excess mortality attributed to influenza (observed over baseline), while blue areas represent non-influenza mortality. Red stars indicate epidemic seasons dominated by the more severe A/H3N2 influenza viruses [10]. Grey arrows indicate the two periods used in Kwong et al.'s comparative study to evaluate the benefits of universal immunization in Ontario, Canada (1997–2000 and 2000–2004) [9]. Note the less frequent circulation of severe A/H3N2 viruses in the second part of Kwong et al.'s study period, 2000–2004. Trends in influenza burden estimates for these periods are provided for the US and Ontario in Table 1.

Seniors suffer about 90% of the influenza seasonal mortality burden, and rates of hospitalization and death are increasing as the population ages [1]. Studies of national trends in the United States and Italy showed that even after adjusting for population aging and pathogenicity of circulating influenza viruses, vaccine uptake in seniors was not associated with a decline in influenza-related mortality ([2,3]; Figure 1). These disappointing experiences can be explained by the phenomenon of immune senescence, whereby immune response to vaccines declines in the last decades of life [4]. These results—along with a growing understanding that the expected effectiveness of vaccination had been greatly overestimated in seniors [5,6]—have fueled debate over the best strategy to protect high-risk populations [1,7].

In response, the US public health authorities have gradually broadened the recommended target group for vaccination, which now includes all children aged six months to 18 years of age [8]. This new strategy is likely to reduce severe pediatric influenza outcomes, and it could also protect seniors by reducing influenza transmission from high-transmitter populations, although the exact benefits have yet to be evaluated.

**Linked Research Article**

This Perspective discusses the following new studies published in *PLoS Medicine*:

Kwong JC, Stukel TA, Lim J, McGeer AJ, Upshur REG, et al. (2008) The effect of universal influenza immunization on mortality and health care use. PLoS Med 5(10): e211. doi:10.1371/journal.pmed.0050211

Comparing influenza-related mortality and health care use between Ontario and other Canadian provinces, Jeffrey Kwong and colleagues find evidence that Ontario's universal vaccination program has reduced the burden of influenza.

van den Dool C, Bonten MJM, Hak E, Heijne JCM, Wallinga J (2008) The effects of influenza vaccination of health care workers in nursing homes: Insights from a mathematical model. PLoS Med 5(10): e200. doi:10.1371/journal.pmed.0050200

Using a mathematical model to simulate influenza transmission in nursing homes, Carline van den Dool and colleagues find that each additional staff member vaccinated further reduces the risk to patients.

## Evaluation of the Universal Vaccination Program in Ontario

The Ontario province of Canada launched a unique influenza vaccination campaign in October 2000, offering free influenza vaccine to everyone over six months of age through extensive delivery in nontraditional settings, including community centers and shopping malls. In this issue of *PLoS Medicine*, Kwong and colleagues report their evaluation of the impact of this intense and costly strategy on a variety of influenza-related outcomes [9]. The authors compared changes in influenza burden in Ontario before (1997–2000) and after (2000–2004) universal vaccination was implemented—relative to changes in surrounding Canadian provinces that had lower vaccine uptake. Electronic health databases and influenza laboratory surveillance data were used to estimate trends in influenza-related mortality, hospitalization, and visits to emergency departments and doctors' offices.

## Study Results

The authors found that the influenza burden decreased as vaccination rates increased across all age groups in Ontario, with a 49%–59% decline for various outcomes (*p* < 0.002), relative to other provinces. Comparisons across provinces showed that there was also a convincing coverage–response relationship among people under 65 years, in that the largest vaccine uptakes corresponded to the greatest reductions in disease burden.

However, the results were more confusing for seniors 65 years and older. Surprisingly, the largest reductions in disease burden in seniors were observed in provinces with the *lowest* vaccine uptakes in this age group. This counterintuitive relationship suggests that changes in vaccine uptake in seniors cannot explain the observed burden reduction in this age group. Further, some of the sensitivity analyses revealed additional unexpected results, including an attenuation of vaccination benefits when focusing on severe influenza seasons or using more data years. In general, however, most sensitivity analyses confirmed the health benefits of the Ontario strategy in all age data, although the age discrepancies were not resolved.

## Strengths and Limitations

Influenza disease burden must be inferred indirectly through statistical methods ([1]; Figure 1), in the face of substantial interannual variations in pathogenicity of circulating influenza viruses, vaccine mismatch seasons, and geographical variability. Thus Kwong and colleagues [9] met great challenges to evaluate the effect of Ontario's population-level experiment. By comparing trends between Ontario and other Canadian provinces and conducting extremely thorough sensitivity analyses, the authors cleverly attempted to control for factors unrelated to vaccine uptake. But even so, lack of statistical power is a key limitation of observational studies comparing disease trends across time periods and geographical regions with different intervention strategies.

To illustrate this methodological problem, if we apply the same approach to US data, we observe large reductions in influenza-related hospitalization and deaths before and after the year 2000 in persons 65 years and older, of similar magnitude to those found in Ontario (Table 1). However, vaccine coverage in US seniors has remained stable in this period ([10]; Figure 1), illustrating that trend analyses need to be interpreted with caution when the study period is short. By contrast, comparison of vaccination and disease trends in people 50–64 years old suggests that larger burden reductions are found in Ontario than in the US, paralleling a larger increase in vaccine uptake in this age group in Ontario. This is an appealing finding, given that numerous randomized clinical trials have reported 70%–90% vaccine efficacy against laboratory-confirmed influenza in younger adults.

A second modeling study presented in this issue of *PLoS Medicine* [11] highlights similar methodological problems in the evaluation of vaccination strategies, and provides insights into the benefits of vaccinating health care workers to protect nursing home residents. Computational simulations of this indirect strategy suggest that, even though increasing the proportion of vaccinated health care workers reduces the risk to nursing home residents, even 100% vaccination coverage cannot entirely eliminate influenza transmission in nursing homes. Further, these simulations reveal that discordances in results of previous clinical studies can be reconciled by taking into account the effects of small sample size and heterogeneity in study settings. In summary, both studies [9,11] illustrate how difficult it is to evaluate the benefits of influenza vaccination strategies, especially in observational settings.

**Table 1:**
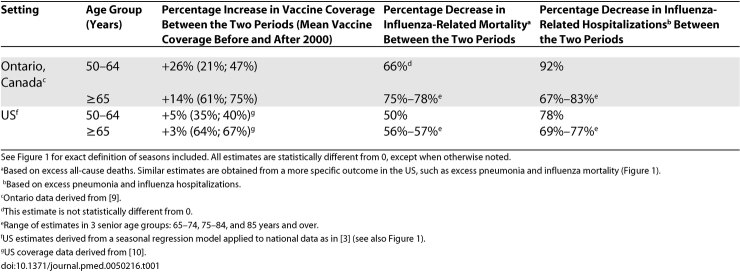
Comparison of Trends in Age-Specific Rates of Influenza-Related Mortality, Hospitalization, and Vaccine Coverage Between Two Periods (1997–2000 and 2000–2004) in the US and Ontario, Canada

## The Way Forward

Based on the experience in Ontario [9] and elsewhere, there is now solid evidence for the effectiveness of influenza vaccination in people under 65 years. However, the direct and indirect benefits of universal immunization remain unclear for seniors, and Kwong and colleagues' study [9] was likely underpowered to evaluate such benefits.

Cluster-randomized trials have been proposed as an alternative and more powerful design than observational studies for evaluating the indirect effects of influenza immunization strategies [12]. The increasing pressure to improve influenza control may eventually generate sufficient interest in sponsoring such large-scale and costly randomized studies. Ironically, there is only a short time window of opportunity for these studies—once pediatric or universal vaccination policy becomes established, there is no ethical “control” community left.

In the meantime, the Ontario intervention program provides valuable information on the benefits of large-scale immunization efforts, and more precise estimates of the direct and indirect benefits in different age groups will likely become available over time. Finally, we note that the Ontario program was very successful in developing innovative channels for influenza vaccine delivery—an experience extremely useful in the context of pandemic preparedness.
